# Outline of Neutron Scattering Formalism

**DOI:** 10.6028/jres.098.002

**Published:** 1993

**Authors:** N. F. Berk

**Affiliations:** National Institute of Standards and Technology, Gaithersburg, MD 20899

**Keywords:** elastic scattering, inelastic scattering, neutron scattering, neutron scattering theory, quasielastic scattering, small angle scattering

## Abstract

Neutron scattering formalism is briefly surveyed. Topics touched upon include coherent and incoherent scattering, bound and free cross-sections, the Van Hove formalism, magnetic scattering, elastic scattering, the static approximation, sum rules, small angle scattering, inelastic scattering, thermal diffuse scattering, quasielastic scattering, and neutron optics.

## 1. Introduction

Neutron scattering provides a direct measure of spatial and temporal correlations of atomic positions and magnetic states of the target system. Thus, to a considerable extent the theory of neutron scattering is aimed at calculating physical quantities that are of interest beyond the scattering technique itself. This outline is a short and highly selective tour through the basic mathematics and concepts of the canonical neutron scattering formalism, which may provide useful preparation for the more focused contributions that follow in this collection. Our approach is didactic but also takes much for granted. We start abruptly, for example, with the general formula for the differential cross section and along the way leave out a great deal that could be said; important topics are ignored, and some of the discussions may seem to end prematurely. If terse, however, the outline also strives to be logical and cohesive and to set out a digestable overview of what is, after all, a far-ranging discipline.

Fortunately for the reader, excellent and well-known introductory articles and texts are available, as well as advanced treatments of both general and specialized interest. References [1–18] of this article serve as a list of some of these. Scherm’s [13] succinct and lively tutorial on the mathematical formalism of neutron scattering was the inspiration for the present treatment, which borrows liberally from it. The opening chapters of Bée’s book [4] also give a thorough and readable survey of the formalism. The introductory review by Price and Sköld [10] has broad scope and provides a balanced development of both theoretical and practical material, while the authoritative collection of articles edited by Sköld and Price [17] reaches into many areas of application. Squires’ clear and efficient textbook [18] is a standard and helpfully incorporates mathematical preparation that other sources often leave out. The treatise by Marshall and Lovesey [8] is a classic presentation of the formalism.

The author is indebted to colleagues who perused drafts of this article at various stages of preparation. Even in its brevity it tries to convey their insightful comments.

## 2. Basic Formulas

### 2.1 The Born Approximation

Our starting point for this outline of neutron scattering formalism is the definition of the double-differential scattering cross-section per particle, as obtained from the Born approximation:

$$\begin{array}{c} {\left(\frac{d^{2}\sigma }{d\Omega d\omega }\right)}_{s_{0}\to s}=\frac{1}{N}{\left(\frac{m}{2\pi \hbar }\right)}^{2}\frac{k}{k_{0}}\sum_{n_{0},n}p\left(n_{0}\right)\\ {\left|\langle k,s,n,\left|V\right|k_{0},s_{0},n_{0}\rangle \right|}^{2}\delta \left(\frac{E_{n}-E_{n_{0}}}{\hbar }-\omega \right), \end{array}$$ (1)

where *Ω* denotes the scattering solid angle, *ω*, the angular frequency corresponding to energy transfer *ħω*, *m*, the neutron mass and *N*, the number of nuclei in the scattering system. Note that in some treatments, the differential cross-section is defined with respect to an energy differential, which introduces a dimensional factor of *ħ*^−1^ on the right-hand side of Eq. (1). The incident neutron beam is in the plane-wave state |***k***_0_, *s*_0_〉, where ***k***_0_ is the incident wavevector and *s*_0_ is the incident spin. The scattered beam is in the state |***k***, *s*〉, where ***k*** and *s* refer to the outgoing beam. The fundamental formula in Eq. (1) effectively is the product of a kinematical factor, *k/k*_0_, with the transition rate from Fermi’s golden rule. The matrix element of the neutron-scatterer interaction *V* is taken in the product representation

|k,s,n〉=|k,s〉|n〉,

where

$$H|n\rangle =E_{n}|n\rangle$$

and *H* is the Hamiltonian of the scatterer. The summation over the initial states of the scatterer is weighted by a probability density, *p* (*n*_0_). The momentum transferred *to* the scatterer by the neutron beam is *ħ****Q***, where

$$Q=k_{0}-k$$ (2)

is called the scattering wavevector, or, more often than not, simply the scattering “vector.” We designate the energies of an incident and scattered neutron as *E*_0_ and *E*, respectively. Then, consistent with the right hand side of Eq. (1), the angular frequency *ω* is determined by the neutron energy, *E*_0_ − *E*, transferred *to* the scatterer:

$$\hbar \omega =\frac{\hbar^{2}}{2m}\left(k_{0}^{2}-k^{2}\right).$$ (3)

Thus positive *ω* corresponds to excitation of the target, which is convenient in calculations which focus on the properties of the scatterer rather than on the analysis and configuration of the measuring instrument. Indeed, with respect to the probing neutron, *ω* > 0 means energy *loss* or “down” scattering, while *ω* < 0 represents energy *gain* or “up” scattering.

### 2.2 Nuclear Scattering in Homogeneous Systems

For nuclear scattering the interaction potential is well-approximated by the Fermi pseudopotential

$$V\left(r\right)=\frac{2\pi \hbar^{2}}{m}\sum_{j}b_{j}\delta \left(r-R_{j}\right),$$ (4)

where *b_j_* is the isotope- and spin-dependent scattering length of the neutron-nuclear interaction, and the summation is over all of the nuclei in the system. The scattering lengths in Eq. (4) are defined for fixed nuclei in the laboratory frame, and are generally referred to as “bound “ (or “boundatom”) scattering lengths. For unbound nuclei, the bound scattering lengths are replaced by “free” scattering lengths,

$$a_{i}=\frac{A_{i}}{A_{i}+1}b_{i},$$ (5)

where the *A_i_* are the nuclear atomic weights. The prefactor in Eq. (5) stems from the reduced mass of the interacting neutron-nucleus pair. The bound scattering lengths, in fact, are always the correct choice, provided the scattering is calculated correctly, as illustrated below by the case of the ideal gas. Thus tabulations of scattering lengths (e.g., the comprehensive table by Sears [14]) give the bound values. For most nuclei, of course, the differences between the bound and free scattering lengths are relatively small, but hydrogen and deuterium are notable exceptions.

For a neutron beam of arbitrary spin polarization, the scattered intensity is determined by

$$\frac{d^{2}\sigma }{d\Omega d\omega }=\sum_{s_{0},s}p\left(s_{0}\right){\left(\frac{d^{2}\sigma }{d\Omega d\omega }\right)}_{s_{0}\to s}$$ (6)

where $p\left(\frac{1}{2}\right)-p\left(-\frac{1}{2}\right)$ is the polarization state of the beam. For an unpolarized beam, $p\left(\pm \frac{1}{2}\right)=\frac{1}{2},$ while for nonmagnetic interactions the cross-section is independent of *s*_0_. When there is no knowledge connecting the internal (isotopic and spin) states of the scattering nuclei to their positions, the observed cross-section is an average over the nuclear degrees of freedom:

$$\frac{d^{2}\sigma }{d\Omega d\omega }={\langle \frac{d^{2}\sigma }{d\Omega d\omega }\rangle }_{\mathrm{nuc}}.$$ (7)

It is generally an excellent approximation to assume that there are no correlations between the internal states of different nuclei. As a consequence,

$$\bar{b_{i}b_{j}}=\bar{b_{i}}\bar{b_{j}},\;i\neq j,$$

where the overbar denotes the average over nuclear internal degrees of freedom. Then

$$\bar{b_{i}b_{j}}=b_{\mathrm{inc}}^{2}\delta_{ij}+b_{\mathrm{coh}}^{2}\;,$$ (8)

where

$$\begin{array}{l} b_{\mathrm{coh}}=\bar{b,}\;\mathrm{and}\\ b_{\mathrm{inc}}^{2}=\bar{b^{2}}-{\bar{b}}^{2}\;, \end{array}$$ (9)

define the interactions for nuclear *coherent* and *incoherent* scattering. The effective scattering lengths, *b*_inc_ and *b*_coh_, are often expressed in terms of bound cross-sections as,

$$\sigma_{\alpha }=4\pi b_{\alpha }^{2},$$ (10)

where

$$\alpha =\{\begin{array}{c} “\mathrm{coh}”,\\ “\mathrm{inc}”. \end{array}$$ (11)

Therefore,

$$\sigma_{\mathrm{inc}}+\sigma_{\mathrm{coh}}=4\pi \bar{b^{2}}.$$ (12)

Equation (8) can be rewritten even more compactly as

$$\bar{b_{i}b_{j}}=\sum_{\alpha }\xi_{i,j}^{\alpha }b_{\alpha }^{2}\;,$$ (13)

where

$$\xi_{i,j}^{\alpha }=\{\begin{array}{c} 1\;\mathrm{for}\;\alpha =“\mathrm{coh}”,\\ \delta_{i,j}\;\mathrm{for}\;\alpha =“\mathrm{inc}”. \end{array}$$ (14)

The combination of Eqs. (1), (4), (7), (13) and (14) then gives

$$\frac{d^{2}\sigma }{d\Omega d\omega }=\frac{k}{4\pi k_{0}}\sum_{\alpha }\sigma_{\alpha }S_{\alpha }\left(Q,\omega \right),$$ (15)

where

$$\begin{array}{l} S_{\alpha }\left(Q,\omega \right)=\frac{1}{N}\sum_{n_{0},n}p\left(n_{0}\right)\sum_{i,j}\xi_{i,j}^{\alpha }\\ \langle n_{0}|e^{-iQ\cdot R_{j}}|n\rangle \langle n|e^{iQ\cdot R_{i}}|n_{0}\rangle \delta \left(\frac{E_{n}-E_{n_{0}}}{\hbar }-\omega \right). \end{array}$$ (16)

Explicitly, these are:

$$\begin{array}{l} S_{\mathrm{coh}}\left(Q,\omega \right)=\frac{1}{N}\sum_{n_{0},n}p\left(n_{0}\right)\sum_{i,j}\\ \langle n_{0}|e^{-iQ\cdot R_{j}}|n\rangle \langle n|e^{iQ\cdot R_{i}}|n_{0}\rangle \delta \left(\frac{E_{n}-E_{n_{0}}}{\hbar }-\omega \right), \end{array}$$ (17)

and

$$\begin{array}{l} S_{\mathrm{inc}}\left(Q,\omega \right)=\frac{1}{N}\sum_{n_{0},n}p\left(n_{0}\right)\sum_{i}\\ {\left|\langle n|e^{iQ\cdot R_{j}}|n_{0}\rangle \right|}^{2}\;\delta \left(\frac{E_{n}-E_{n_{0}}}{\hbar }-\omega \right). \end{array}$$ (18)

Notice that the site summation in *S*_coh_ (***Q***, *ω*) is unrestricted and thus includes the summation in *S*_inc_ (***Q***, *ω*). The total cross-section is obtained from

$$\sigma_{\mathrm{total}}=\int \int_{-\infty }^{E_{0}/h}\frac{d^{2}\sigma }{d\Omega d\omega }d\Omega d\omega .$$ (19)

In the upper limit, $E_{0}=\hbar^{2}k_{0}^{2}/2m$ is the incident energy, the largest possible neutron energy loss. Often, however, *ω*-integral is well-approximated by taking the upper limit to ∞. The quantity

$$\sum =\frac{N}{V}\sigma_{\mathrm{total}},$$ (20)

for *N* scattering nuclei in sample volume *V*, is called the *macroscopic* cross-section and is just the inverse of the scattering mean free path.

The total cross-section, Eq. (19), depends on the incident neutron energy; note, for example, the factor $k_{0}=\sqrt{2m-E_{0}}/\hbar$ in Eq. (1). The *E*_0_-dependence of total cross-section can be obtained analytically for the case of neutron scattering by an ideal gas. The result shows that $\sigma_{\mathrm{total}}\propto E_{0}^{-1/2}\;\mathrm{for}\;E_{0}\to 0$, as expected from the kinematics, while for *E*_0_ ≫ *k*_B_*T/A*, the asymptotic scattering is

$$\sigma_{\mathrm{total}}={\left(\frac{A}{A+1}\right)}^{2}\left(\sigma_{\mathrm{inc}}+\sigma_{\mathrm{coh}}\right),$$

which is just the free cross-section. This renormalization of the bound cross-sections at high incident energy is brought about by the recoil kinetic energy of the impulsive neutron-atom collisions and is observed in condensed matter.

The relation in Eq. (15) is the basic law of nonmagnetic neutron scattering from homogeneous systems. Generalizations to heterogeneous nuclear scattering and magnetic scattering, described below, depart only slightly from this archetype. Thus (nuclear) neutron scattering is a sum of *coherent* and *incoherent* contributions, each of which conveys different kinds of information about the scattering system. Only *S*_coh_(***Q***, *ω*) expressly depends on the relative positions and motions of the nuclei and thus provides explicit knowledge of structure and collective dynamics. In contrast, *S*_inc_(***Q***, *ω*) discloses no spatial structure and only reveals the motions of individual particles. For example, while coherent scattering can measure a phonon dispersion curve, *ω* vs ***Q***, incoherent scattering can only measure a phonon density of states, *N* (*ω*). Needless to say, a density of states is often just the information desired.

The weights of coherent and incoherent scattering in the measurement are determined — for a homogeneous system — by *σ*_coh_ and *σ*_inc_, which vary randomly from one nuclear species to another. Most nuclei are stronger coherent scatterers than incoherent scatters, and a few even scatter *only* coherently. For example, if we define

$$f_{\alpha }=\frac{\sigma_{\alpha }}{\sigma_{\mathrm{total}}},$$ (21)

then *f*_coh_ = 1 for the abundant isotopes of Al, C, and O, while *f*_coh_ ≥ 0.97 for Mg and Fe. Some nuclei scatter mostly *incoherently*. For example, *f*_inc_(V) = 0.995 and *f*_inc_(Co) = 0.84. Hydrogen, *f*_inc_(H) = 0.98, is a singular example of an incoherent scatterer. Its total cross-section, *σ*_total_(H) = 82 barns (1 barn = 10^−28^m^2^), is by far the largest among the elements and, of course, it is abundant in many materials. Hydrogen in hydrated compounds and in hydrocarbons causes significant incoherent scattering, which often masks the desired coherent scattering in structure determinations. Indeed, in small angle neutron scattering, water scattering, which is virtually independent of ***Q*** in the range of interest, is used to calibrate area detectors for spatial nonuniformity. Deuterium scatters incoherently, as well, but actually is a stronger coherent scatterer, with *f*_coh_(*D*) = 0.74. Hydrogen and deuterium also are readily dissolved in many metals as a highly mobile interstitial impurity. Incoherent neutron scattering thus probes hydrogen vibrations and transport in these metals: in *S*_inc_(***Q***, *ω*), the position operator, ***R***_i_ (*t*), then refers to only to H if the host metal is a coherent scatterer.

In heterogeneous nuclear systems, Eq. (15) is replaced by

$$\frac{d^{2}\sigma }{d\Omega d\omega }=\frac{k}{k_{0}}\sum_{\alpha }S_{\alpha }^{\mathrm{het}}\left(Q,\omega \right),$$ (22)

where

$$\begin{array}{l} S_{\alpha }^{\mathrm{het}}\left(Q,\omega \right)=\frac{1}{N}\sum_{n_{0},n}p\left(n_{0}\right)\sum_{i,j}\Xi_{i,j}^{\alpha }\\ \langle n_{0}|e^{-iQ\cdot R_{j}}|n\rangle \langle n|e^{iQ\cdot R_{j}}|n_{0}\rangle \delta \left(\frac{E_{n}-E_{n_{0}}}{\hbar }-\omega \right), \end{array}$$ (23)

and where the *ξ*-function defined in Eq. (14) has been extended to the *Ξ*-function,

$$\Xi_{i,j}^{\alpha }=\{\begin{array}{c} b_{\mathrm{coh},i}\;b_{\mathrm{coh},j}\;\mathrm{for}\;\alpha =“\mathrm{coh}”,\\ \delta_{i,j}b_{\mathrm{inc},i}^{2}\;\mathrm{for}\;\alpha =“\mathrm{inc}”. \end{array}$$ (24)

Thus, in heterogeneous systems, including crystals with bases, the coherent scattering is also sensitive to the *signs* of the constituent *b*_coh_-values, which also vary randomly with species, although *b*_coh_ > 0 in most cases. For example *b*_coh_(H) = −0.37, while *b*_coh_(D) = 0.65. This sign difference is exploited in a wide variety of neutron scattering experiments, in which deuteration is used to label molecular sites or adjust overall coherent scattering contrasts to enhance the contributions from various components of the structure.

### 2.3 Van Hove Formalism

The Van Hove formalism recasts the Born approximation formula for *S_α_*(***Q***, *ω*) into the Heisenberg representation and introduces quantum generalizations of classical correlation functions. Begin with the identity,

$$\begin{array}{l} \langle m|A_{\mathrm{op}}\delta \left(\frac{E_{n}-E_{m}}{\hbar }\right)|n\rangle =\\ \;\frac{1}{2\pi }\int_{-\infty }^{\infty }e^{i\omega t}\langle m|A_{op}\left(t\right)|n\rangle dt, \end{array}$$ (25)

where

$$A_{\mathrm{op}}\left(t\right)=e^{i\frac{H}{\hbar }t}A_{\mathrm{op}}e^{-i\frac{H}{\hbar }t}.$$ (26)

Then Eq. (16) becomes

$$S_{\alpha }\left(Q,\omega \right)=\frac{1}{2\pi }\int_{-\infty }^{\infty }e^{-i\omega t}I_{\alpha }\left(Q,t\right)dt,$$ (27)

with the introduction of

$$I_{\alpha }\left(Q,t\right)=\frac{1}{2}\sum_{i,j}\xi_{i,j}^{\alpha }\langle e^{-iQ\cdot R_{i}\left(0\right)}e^{-iQ\cdot R_{j}\left(t\right)}\rangle .$$ (28)

Two additional steps were also needed to reach Eq. (28). First

$$e^{i\frac{H}{\hbar }t}e^{iQ\cdot R}e^{-i\frac{H}{\hbar }t}=e^{-iQ\cdot R\left(t\right)}$$

and

$$\sum_{n_{0}}p\left(n_{0}\right)\langle n_{0}|A_{\mathrm{op}}|n_{0}\rangle =\langle A_{\mathrm{op}}\rangle .$$

Finally, with the use of the obvious identity,

$$e^{iQ\cdot R_{j}}=\int e^{iQ\cdot r_{j}}\delta \left(r-R_{j}\right)d^{3}r,$$

Eq. (28) takes the form

$$I_{\alpha }\left(Q,t\right)=\int e^{iQ\cdot r_{j}}G_{\alpha }\left(r,t\right)d^{3}r.$$ (29)

where

$$\begin{array}{l} G_{\alpha }\left(r,t\right)=\frac{1}{N}\sum_{i,j}\;\xi_{i,j}^{\alpha }\int \\ \;\langle \delta \left(r^{\prime }-R_{i}\left(0\right)\right)\delta \left(r^{\prime }+r-R_{j}\left(t\right)\right)\rangle d^{3}r^{\prime }. \end{array}$$ (30)

Then *S_α_*(***Q***, *ω*) and *G_α_*(***r***, *t*) are connected by the Fourier transform

$$\begin{array}{l} S_{\alpha }\left(Q,\omega \right)=\frac{1}{2\pi }\int \int_{-\infty }^{\infty }\\ \;e^{i\left(Q\cdot r-\omega t\right)}G_{\alpha }\left(r,t\right)d^{3}rdt, \end{array}$$ (31)

and its inverse,

$$\begin{array}{l} G_{\alpha }\left(r,t\right)=\int \int_{-\infty }^{\infty }\\ \;e^{-i\left(Q\cdot r-\omega t\right)}S_{\alpha }\left(Q,\omega \right)\frac{d^{3}Q}{{\left(2\pi \right)}^{3}}d\omega \;. \end{array}$$ (32)

The asymmetric placement of the 2*π* in these Fourier integrals is unfortunate, perhaps, but conventional. It could be avoided, say, by multiplying the right-hand-side of Eq. (28) by 2*π*, but this is never done. The relationships among the quantities

*S_α_*(***Q***,*ω*)*→* “scattering function”,
*I_α_*(***Q***,*t*)→ “intermediate scattering function”,
*G_α_*(***r***,*t*)→ “correlation function”,

comprise the Van Hove representation of the Born approximation. Now the nuclear position operators, ***R****_i_*(*t*), are not constants of motion, so that

$$\left[R_{i}\left(t\right),R_{j}\left(t^{\prime }\right)\right]\neq 0,$$

and the substitutions implied by the the δ-functions can not be made in Eq. (30), except in a classical approximation. However, the correlation function in the coherent case can be exactly represented by the form

$$G_{\mathrm{coh}}\left(r,t\right)=\frac{1}{N}\int \langle \rho \left(r^{\prime },0\right)\rho \left(r^{\prime }+r,t\right)\rangle d^{3}r^{\prime },$$ (33)

where

$$\rho \left(r,t\right)=\sum_{i}\;\delta \left(r-R_{i}\left(t\right)\right)$$ (34)

is the nuclear particle density operator. Also, with

$$\rho \left(Q,t\right)=\int e^{iQ\cdot r}\rho \left(r,t\right)d^{3}r,$$ (35)

the coherent intermediate scattering function is also represented by

$$I_{\mathrm{coh}}\left(Q,t\right)=\frac{1}{N}\langle \rho \left(-Q,0\right)\rho \left(Q,t\right)\rangle .$$ (36)

In materials applications, the meaning of 〈…〉 is generalized to include configurational averaging, as well as the implied thermal average. For a heterogeneous scattering population, *b*_coh_*p*(***r***,*t*) becomes the *scattering-length density* field,

$$\eta \left(r,t\right)=\sum_{i}b_{\mathrm{coh},i}\delta \left(r-R_{i}\left(t\right)\right),$$ (37)

and $S_{\mathrm{coh}}^{\mathrm{het}}\left(Q,\omega \right)$ is a sum of contributions depending on self- and cross-correlations among the various nuclear populations.

The Van Hove formulation was a signal advance in non-relativistic scattering theory and a major stimulus to condensed matter applications beyond crystallography. Although rooted in Fermi’s golden rule, it manages, with the introduction of the correlation function, to “hide” its overt quantum mechanical origins and bring forth a classical looking representation that is a powerful guide to intuition, while also providing the path to relationships with statistical mechanics and linear response theory.

In thermal equilibrium, the statistical weight *p*(*n*_0_) of the initial states is the Boltzmann factor

$$p\left(n_{0}\right)=\frac{e^{-\beta En0}}{Z}$$

where

$$\beta =\frac{1}{k_{B}T},\;\mathrm{and}\;Z=\sum_{n}e^{-\beta En}.$$

The energy conservation explicit in Eq. (1) means that

$$p\left(n_{0}\right)\to p\left(n\right)e^{\beta \hbar \omega }.$$

within the sums over states. This leads eventually to the *principal of detailed balance*:

$$S_{\alpha }\left(-Q,-\omega \right)=e^{-\beta \hbar \omega }S_{\alpha }\left(Q,\omega \right).$$ (38)

In applications to inelastic scattering, Eq. (38) predicts that “down” scattering (*ω*>0) is always stronger than “up” scattering (*ω*<0). For centro-symmetric systems, the scattering function is an even function of ***Q***,

$$S_{\alpha }\left(-Q,\omega \right)=S_{\alpha }\left(Q,\omega \right).$$ (39)

Actually, this is also true for systems without a center of symmetry if the inverted equililbrium structure differs from the original by a constant displacement.

The scattering function, *S_α_*(***Q***,*ω*), is defined to be real, but the derived correlation function, *G_α_*(***r***,*t*), is complex:

$$G\left(r,t\right)=G^{\prime }\left(r,t\right)+iG^{\prime \prime }\left(r,t\right).$$

This can be seen from the integral in Eq. (32), since *S_α_*(***Q***,*ω*) is not an even function of *ω*—the constraint of detailed balance. Thus a general relationship exists between the real and imaginary parts of the correlation function, which ultimately leads to:

$$\begin{array}{l} S_{\alpha }\left(Q,\omega \right)=\frac{2i}{1-e^{-\beta \hbar \omega }}\\ \;\int e^{\left(iQ\cdot r-\omega t\right)}G_{\alpha }^{\prime \prime }\left(r,t\right)d^{3}rdt. \end{array}$$ (40)

This leads in turn to a concise connection between the scattering theory and linear response theory:

$$S_{\mathrm{coh}}\left(Q,\omega \right)=\frac{-2\hbar }{1-e^{-\beta \hbar \omega }}\chi^{\prime \prime }\left(Q,\omega \right),$$ (41)

where *χ*(***Q***,*ω*) is the generalized susceptibility function for the density operator in Eq. (34).

### 2.4 Magnetic Scattering of Unpolarized Neutrons

Since the neutron has a magnetic moment, incident neutrons interact not only with the nuclei of the atoms in the scatterer but also with the magnetic moments of their unpaired electrons. For unpolarized beams, the magnetic interaction with the nuclear spins is already incorporated into the distinction between *b*_coh_ and *b*_inc_ in Eq. (9). The potential for the neutron-electron interaction is

$$V_{\mathrm{mag}}\left(r\right)=-\mu \cdot B\left(r\right),$$ (42)

where *μ* = *γμ*_N_*σ* is the neutron magnetic moment operator, in terms of the neutron gyromagnetic ration, *γ* = 1.9132, the nuclear magneton, *μ*_N_, and the Pauli operator, *σ*. The internal magnetic field operator, ***B***(***r***), is

$$B\left(r\right)=2\mu_{B}\sum_{i}\left[\nabla \times \left(\frac{s_{i}\times \hat{r}}{r^{2}}\right)+\frac{p_{i}\times \hat{r}}{r^{2}}\right],$$ (43)

where *μ*_B_ is the Bohr magneton, ***s****_i_* and ***p****_i_* are the electron spin and momentum operators, respectively, and the summation is over all unpaired electrons. The (^) symbol denotes a unit vector. For the Born approximation the required matrix element of ***B***(***r***) is

$$\langle k|B\left(r\right)|k_{0}\rangle =2\mu_{B}\sum_{i}\left[\hat{Q}\times \left(s_{i}\times \hat{Q}\right)+\frac{i\left(p_{i}\times \hat{Q}\right)}{\hbar Q}\right]e^{iQ\cdot r}$$ (44)

$$=\mu_{0}M\left(Q\right),$$ (45)

where *μ*_0_ is the vacuum magnetic permeablity and ***M*** is the magnetization operator. For an unpolarized beam, the differential cross-section can be expressed as in Eq. (15) with the identifier *α* taking on the additional value “mag”, and with the definition

$$\sigma_{\mathrm{mag}}=4\pi {\left(\gamma r_{0}\right)}^{2},$$ (46)

where *r*_0_ is the classical electron radius. Note that this usage of the symbol, *σ*_mag_, is not universal. The generalization of the Van Hove representation to the magnetic case gives,

$$\begin{array}{l} S_{\mathrm{mag}}\left(Q,\omega \right)=\sum_{\mu ,\upsilon }\frac{\delta_{\mu \upsilon }-{\hat{Q}}_{\mu }{\hat{Q}}_{\upsilon }}{{\left(2\mu_{B}\mu_{0}\right)}^{2}}\frac{1}{2\pi }\\ \;\int_{-\infty }^{\infty }I_{\mu \upsilon }^{\mathrm{mag}}\left(Q,t\right)dt, \end{array}$$ (47)

where *μ* and *υ* are coordinate labels. The intermediate scattering function $I_{\mu \upsilon }^{\mathrm{mag}}\left(Q,t\right)$ extends the definition in Eq. (36),

$$I_{\mu \upsilon }^{\mathrm{mag}}\left(Q,t\right)=\frac{1}{N_{m}}\langle M_{\mu }\left(-Q,0\right)M_{\upsilon }\left(Q,t\right)\rangle ,$$ (48)

for *N*_m_ magnetic ions, while the corresponding magnetization correlation function, from Eq. (33), is

$$G_{\mu \upsilon }^{\mathrm{mag}}\left(r,t\right)=\frac{1}{N_{m}}\int \langle M_{\mu }\left(r^{\prime },0\right)M_{\upsilon }\left(r^{\prime }+r,t\right)\rangle d^{3}r^{\prime }.$$ (49)

Thus, except for the appearance of the quantity known as the Halpern tensor,

$$\delta_{\mu \upsilon }-{\hat{Q}}_{\mu }{\hat{Q}}_{\upsilon },$$ (50)

in Eq. (47), the formal extension of nuclear scattering to magnetic scattering of unpolarized beams simply entails replacing the scalar nuclear density, *ρ*, by the components of the magnetization, ***M***_μ_. The Halpern factor, however, is responsible for the special, anisotropic character of magnetic neutron scattering. For example, for a single-domain ferromagnetic sample,

$$\delta_{\mu \upsilon }-{\hat{Q}}_{\mu }{\hat{Q}}_{\upsilon }\to 1-{\left(\hat{Q}\cdot \hat{M}\right)}^{2}$$ (51)

where $\hat{M}$ is the direction of the domain magnetization. Thus, in the Born approximation, there is no magnetic scattering for ***Q*** ‖ ***M***. As indicated by the scattering law, Eq. (15), now with

$$\alpha =\{\begin{array}{c} \;“\mathrm{coh}”,\\ “\mathrm{inc}”,\\ \;“\mathrm{mag}”, \end{array}$$ (52)

nuclear and magnetic scattering coexist, in general, in magnetic materials and can have comparable weight. The anisotropy of magnetic scattering offers one means of separating these contributions by choosing directions of ***Q*** along which the magnetic component is suppressed. Squires [18] introduces basic elements of magnetic scattering, while Balcar and Lovesey [3] provide a thorough exposition.

## 3. Generic Applications

### 3.1 Elastic Scattering

The scattering function can be analyzed into distinct *ω*-dependent contributions according to:

$$S_{\alpha }\left(Q,\omega \right)=I_{\alpha }\left(Q,\infty \right)\delta \left(\omega \right)+S_{\alpha }^{\mathrm{ne}}\left(Q,\omega \right).$$ (53)

The first part is called the *elastic* scattering, which, by convention, means scattering with no change in the neutron energy, so that *k* = *k*_0_. The isolation of the elastic contribution is the result of writing

$$I_{\alpha }\left(Q,t\right)=I_{\alpha }\left(Q,\infty \right)+\left[I_{\alpha }\left(Q,t\right)-I_{\alpha }\left(Q,\infty \right)\right],$$ (54)

in the integrand of Eq. (27) and assuming that the limit,

$$I_{\alpha }\left(Q,\infty \right)=\underset{t\to \infty }{\lim}\left[I_{\alpha }\left(Q,t\right)\right],$$

exists. The first term in Eq. (54) is independent of *t* and thus produces the scattering proportional to *δ*(*ω*). The non-elastic part $S_{\alpha }^{\mathrm{ne}}\left(Q,\omega \right)$ stems from the Fourier transform of the *t*-dependent remainder and comprises both *in*elastic and *quasi*elastic contributions. Furthermore, for |*t*−*t*′|→ ∞,

$$\begin{array}{l} \langle A\left(t\right)B\left(t^{\prime }\right)\rangle =\langle A\left(t\right)\rangle \langle B\left(t^{\prime }\right)\rangle \\ \;=A^{\mathrm{eq}}B^{\mathrm{eq}}, \end{array}$$ (55)

where the *A*^eq^ is equilibrium average of the operator *A*. Therefore, in Eq. (53)

$$I_{\mathrm{coh}}\left(Q,\infty \right)=\frac{1}{N}{\left|\rho^{\mathrm{eq}}\left(Q\right)\right|}^{2},$$ (56)

which is the basis of structure determinations in solids. Various aspects of this extensive field are elucidated in [1], [7], [8], [11], and [18]. For liquids, on the other hand, *ρ*^eq^(***r***)=*N/V*, so that

$$I_{\mathrm{coh}}\left(Q,\infty \right)=\frac{1}{N}{\left(\frac{N}{V}\right)}^{2}\delta \left(Q\right),$$

leading to the dictum that liquids scatter elastically only in the forward direction. The weight of incoherent elastic scattering is given by

$$I_{\mathrm{inc}}\left(Q,\infty \right)=\int e^{iQ\cdot r}P_{\mathrm{self}}\left(r,\infty \right)dr,$$ (57)

where

$$P_{\mathrm{self}}\left(r\right)=\int \langle \delta \left(r^{\prime }\right)\rangle \langle \delta \left(r^{\prime }+r\right)\rangle d^{3}r^{\prime }$$ (58)

can be interpreted as the conditional probability density for nuclear position ***r*** at time *t*→∞, given that ***r*** = **0** at *t* = 0. These expressions for the elastic scattering are easily extended to heterogeneous systems.

### 3.2 Energy-Integrated Scattering: The Static Approximation

Coherent elastic scattering, which reveals equilibrium structure, must be measured by energy-resolved techniques. Often, however, structure is studied using methods that do not discriminate the scattered neutron energy; these experiments observe the energy-integrated cross-section

$$\frac{d\sigma }{d\Omega }=\int_{-\infty }^{\frac{E_{0}}{\hbar }}\frac{d^{2}\sigma }{d\Omega d\omega }d\omega .$$ (59)

In the *static approximation*, this is represented as

$${\left(\frac{d\sigma }{d\Omega }\right)}_{\mathrm{static}}=\frac{\sigma_{\mathrm{inc}}}{4\pi }S_{\mathrm{inc}}\left(Q\right)+\frac{\sigma_{\mathrm{coh}}}{4\pi }S_{\mathrm{coh}}\left(Q\right),$$ (60)

where

$$S_{\alpha }\left(Q\right)=\int_{-\infty }^{\infty }S_{\alpha }\left(Q,\omega \right)d\omega .$$ (61)

Since ***Q*** is held fixed in the *ω*-integration, this approximation entails neglecting the ***Q***-dependence of the final neutron wavenumber,

$$k=\left|k_{0}-Q\right|,$$

and setting *k* = *k*_0_, as in elastic scattering. Then from Eq. (27),

$$S_{\alpha }\left(Q\right)=I_{\alpha }\left(Q,0\right).$$ (62)

Since [***R****_i_*(*t*), ***R****_j_*(*t*)] = 0, this can be analyzed as

$$S_{\alpha }\left(Q\right)=\int e^{-iQ\cdot r}G_{\alpha }\left(r,0\right)d^{3}r,$$ (63)

where

$$G_{\alpha }\left(r,0\right)=\frac{1}{N}\sum_{i,j}\;\xi_{i,j}^{\alpha }\langle \delta \left(r+R_{i}\left(0\right)-R_{j}\left(0\right)\right)\rangle .$$ (64)

Therefore, for the incoherent contribution,

$$G_{\mathrm{inc}}\left(r,0\right)=\delta \left(r\right),$$ (65)

so that

$$S_{\mathrm{inc}}\left(Q\right)=1.$$ (66)

For the more interesting coherent component,

$$S_{\mathrm{coh}}\left(Q\right)=\delta \left(r\right)+g\left(r\right),$$ (67)

where

$$g\left(r\right)=\sum_{j\neq 0}\langle \delta \left(r+R_{0}\left(0\right)-R_{j}\left(0\right)\right)\rangle$$ (68)

is the static pair-correlation function. Thus energy-integrated coherent scattering measures an average of instantaneous “snapshots” of the time-dependent structure, while elastic coherent scattering measures the equilibrium averaged structure. In harmonic crystalline solids, where the atoms more-or-less “stay put,” both methods yield the equilibrium positions and mean-square thermal displacements, but the two forms of measurement differ in important ways. Indeed, from Eq. (53), one has

$$S_{\mathrm{coh}}\left(Q\right)=S_{\mathrm{coh}}^{\mathrm{el}}\left(Q\right)+S_{\mathrm{coh}}^{\mathrm{ne}}\left(Q\right),$$ (69)

where we have defined

$$S_{\mathrm{coh}}^{\mathrm{el}}\left(Q\right)=I_{\mathrm{coh}}\left(Q,\infty \right),$$ (70)

and

$$S_{\mathrm{coh}}^{\mathrm{ne}}\left(Q\right)=\int_{-\infty }^{\infty }S_{\mathrm{coh}}^{\mathrm{ne}}\left(Q,\omega \right)d\omega .$$ (71)

For monatomic harmonic crystals, in particular,

$$S_{\mathrm{coh}}^{\mathrm{el}}\left(Q\right)=\frac{{\left(2\pi \right)}^{3}}{\Omega_{0}}e^{-2w}\sum_{k}\;\delta \left(Q-k\right),$$ (72)

where *Ω*_0_ is the volume of the unit cell, the *K* are wavevectors of the reciprocal lattice, and exp(−2*W*) is the Debye-Waller factor, which depends on the thermal displacements and is defined below at Eq. (107). The *δ*-functions in Eq. (72) are the Bragg peaks from which the equilibrium structure is deduced. In this context the integrated nonelastic scattering in Eq. (69) is referred to as *thermal diffuse scattering*. For the special case of an Einstein solid (dispersionless vibrations), the thermal diffuse scattering varies monotonically with ***Q***. In general, however, for dispersive vibrations (acoustic phonons), the thermal diffuse scattering is a structured background, including temperature-dependent “wings” centered on the Bragg peaks. Thermal diffuse scattering is discussed again in Sec. 3.5.2.

In strongly anharmonic solids and systems where structure changes on the time scale of the measurements, elastic and energy-integrated scattering probe substantially different correlations, and only the latter provides the short-time information usually of interest in such cases.

### 3.3 Sum Rules

Taken in a larger context, the formula in Eq. (61) is the zeroth-order member of a set of frequency moments of the scattering function, *S_α_*(***Q***,*ω*), which in normalized form is defined by

$${\langle \omega^{n}\rangle }_{\alpha }\left(Q\right)=\frac{1}{S_{\alpha }\left(Q\right)}\int_{-\infty }^{\infty }\omega^{n}S_{\alpha }\left(Q,\omega \right)d\omega .$$ (73)

In particular,

$${\langle \omega^{0}\rangle }_{\alpha }\left(Q\right)=1.$$ (74)

While seeming trivial, in practice Eq. (74) actually constrains physically distinct measurements and thus provides a means of checking their mutual consistency. The higher-order rules are formally generated starting with Eq. (27):

$$\frac{d^{n}}{dt^{n}}I_{\alpha }\left(Q,t\right){|}_{t=0}=i^{n}\int_{-\infty }^{\infty }S_{\alpha }\left(Q,\omega \right)\omega^{n}d\omega .$$ (75)

Then it is easy to get

$$\begin{array}{l} {\langle \omega^{n}\rangle }_{\alpha }\left(Q\right)=\frac{{\left(-i\right)}^{n}}{NS_{\alpha }\left(Q\right)}\sum_{i,j}\;\xi_{i,j}^{\alpha }\\ \;{\langle e^{-iQ\cdot R_{j}\left(0\right)}\frac{d^{n}}{dt^{n}}e^{iQ\cdot R_{j}\left(t\right)}\rangle }_{t=0}, \end{array}$$ (76)

where, following Eq. (26), the derivatives with respect to *t* are determined by

$$\frac{d}{dt}e^{iQ\cdot R_{j}\left(t\right)}=\frac{i}{\hbar }\left[H,e^{iQ\cdot R_{j}\left(t\right)}\right].$$ (77)

For velocity-independent interactions, the commutator is independent of the atom-atom interaction potential and gives

$$\begin{array}{l} \left[H,e^{iQ\cdot R_{j}\left(t\right)}\right]=\left[-\frac{\hbar^{2}\nabla_{j}^{2}}{2M},e^{iQ\cdot R_{j}\left(t\right)}\right]=\\ \;e^{iQ\cdot R_{j}\left(t\right)}\left(\frac{\hbar^{2}Q^{2}}{2M}+\hbar Q\cdot R_{j}\left(t\right)\right), \end{array}$$ (78)

where *M* is the atomic mass. The contribution from the second term averages to zero in Eq. (76), and, recalling the normalization in Eq. (73), it follows easily that

$$\hbar {\langle \omega \rangle }_{\alpha }\left(Q\right)=\frac{\hbar^{2}Q^{2}}{2M},$$ (79)

which can be interpreted as the average recoil kinetic energy at fixed scattering vector ***Q***. Note that if the *t*-derivative is treated classically, the result is equivalent to keeping only the second term in Eq. (78), which gives 〈*ω*〉*_α_*(***Q***) = 0. This is inconsistent with the asymmetry of *S_α_*(***Q***,*ω*) required by detailed balance, Eq. (38), except in the high-temperature limit.

Higher moments are increasingly system-dependent and more difficult to calculate. For a classical liquid, the second moments are

$${\langle \omega^{2}\rangle }_{\alpha }\left(Q\right)=\frac{Q^{2}k_{B}T}{MS_{\alpha }\left(Q\right)}.$$ (80)

The coherent case,

$${\langle \omega^{2}\rangle }_{\mathrm{coh}}\left(Q\right)=\frac{Q^{2}k_{B}T}{MS_{\mathrm{coh}}\left(Q\right)},$$ (81)

and its analogues come into play in the analysis of quasielastic scattering (Sec. 3.6) in liquids and lattice fluids, where 〈*ω*^2^〉_coh_(***Q***) is used as a measure of the width of the coherent contribution to the quasielastic line. Then Eq. (81) predicts that the width has a minimum near the maximum of *S*_coh_(*Q*), the effect known as *de Gennes narrowing*, which physically expresses the relative stability of density fluctuations that are consonant with the dominant short-ranged liquid order at *Q*_max_.

### 3.4 Small Angle Neutron Scattering (SANS)

Small angle scattering is the discipline of studying microstructure of materials by investigating scattering in the immediate neighborhood of the incident beam. For neutrons with wavelengths larger than the *Bragg cutoff* — i.e., *λ*_0_ > *α*, where *a* is the largest unit cell dimension—Bragg scattering from the atomic-scale structure is suppressed, and all scattering is near the forward direction. The *Q*-dependence of small angle scattering thus arises from structural variations over length scales usually much larger than the atomic scale. An informal but helpful rule is that microstructure on length scale *L* produces scattering in the *Q*-range

$$Q\leq 2\pi L^{-1}.$$ (82)

In other words, the more homogeneous the microstructure, the smaller the *Q*-range in which it is observable. To a good approximation in SANS, the scattering angle is given by 2*θ* = *λ*_0_*Q*/2*π*. Combining this with the criterion in Eq. (82), gives

$$2\theta \leq \frac{\lambda_{0}}{L}.$$ (83)

This shows that increasing the neutron wavelength magnifies the observable scale of microstructure; i.e., at a given scattering angle (position on the detector relative to the incident beam), larger homogeneous regions contribute to the scattering at larger incident wavelengths. Small angle scattering also occurs for neutron wavelengths below the Bragg cutoff, but then intensity at small angles is reduced by Bragg scattering into large angles. In thick specimens, especially, multiple Bragg scattering can contaminate scattering in the small angle regime, a bane of the early history of the field.

As with other methods of structure determination, small angle scattering can be implemented as elastic scattering, following Eqs. (53) and (56), or as energy-integrated scattering, as defined in the preceding section. Most SANS instruments, in fact, integrate the scattered energy transfer, and the static approximation usually is appropriate. Thus, for heterogeneous systems Eqs. (84) and (63) are generalized as

$${\left(\frac{d\sigma }{d\Omega }\right)}_{\mathrm{static}}=\sum_{\alpha }\;S_{\alpha }^{\mathrm{het}}\left(Q\right),$$ (84)

and

$$S_{\alpha }^{\mathrm{het}}\left(Q\right)=\;\int \;e^{iQ\cdot r}G_{\alpha }^{\mathrm{het}}\;\left(r,0\right)d^{3}r.$$ (85)

Since the notation is getting cumbersome, for the remainder of this section the superscript “het” will be omitted, and the specification, *t* = 0, will become implicit. Also, recalling Eq. (9)*b*_coh_ will be denoted by the more compact $\bar{b}$. Now, in order to make contact with conventional SANS terminology, introduce the function *Γ*(***r***),

$$G_{\mathrm{coh}}\left(r\right)=\frac{V}{N}\Gamma \left(r\right).$$ (86)

Then, from Eqs. (33) and (37),

$$\Gamma \left(r\right)=\frac{1}{V}\int \langle \eta \left(r^{\prime }\right)\eta \left(r^{\prime }+r\right)\rangle d^{3}r^{\prime }.$$ (87)

For the incoherent contribution, Eq. (30) easily gives

$$G_{\mathrm{inc}}\left(r\right)=\langle b_{\mathrm{inc}}^{2}\rangle \delta \left(r\right),$$ (88)

where

$$\langle b_{\mathrm{inc}}^{2}\rangle =\frac{1}{N}\sum_{i}b_{\mathrm{inc},i}^{2}.$$ (89)

This is just the extension of Eq. (65) to heterogeneous systems. Small angle scattering usually is analyzed in terms of the Porod-Debye correlation function, as defined by

$$\gamma \left(r\right)=\frac{\Gamma \left(r\right)-\Gamma \left(\infty \right)}{\Gamma \left(0\right)-\Gamma \left(\infty \right)}.$$ (90)

By construction, it follows immediately that

$$\begin{array}{l} \;\gamma \left(0\right)=1,\;\mathrm{and}\\ \gamma \left(\infty \right)=0. \end{array}$$ (91)

Using Eq. (87), the limits of *γ*(***r***) are

$$\begin{array}{l} \;\gamma \left(0\right)=\langle \eta^{2}\rangle v,\;\mathrm{and}\\ \gamma \left(\infty \right)={\langle \eta \rangle }_{v}^{2}, \end{array}$$ (92)

where

$${\langle \ldots \rangle }_{v}=\frac{1}{V}\int \langle \ldots \rangle d^{3}r,$$ (93)

means a combined thermal-configurational-volume average. In most theoretical SANS treatments these averages are treated as being redundant, and the choice of which one to do “first” is usually a matter of mathematical convenience. The Porod-Debye correlation function can then be written as

$$\gamma \left(r\right)=\frac{\frac{1}{V}\int \langle \Delta \eta \left(r^{\prime }\right)\Delta \eta \left(r^{\prime }+r\right)\rangle d^{3}r^{\prime }}{{\langle {\left(\Delta \eta \right)}^{2}\rangle }_{v}},$$ (94)

where

$$\Delta \eta \left(r\right)=\eta \left(r\right)-{\langle \eta \rangle }_{v},$$ (95)

is the fluctuating part of the scattering-length density field. The combination of these results gives

$$\begin{array}{l} N{\left(\frac{d\sigma }{d\Omega }\right)}_{\mathrm{static}}=N\langle b_{\mathrm{inc}}^{2}\rangle +{\left(2\pi \right)}^{3}V{\langle {\left(\Delta \eta \right)}^{2}\rangle }_{v}\delta \left(Q\right)\\ \;+V{\langle {\left(\Delta \eta \right)}^{2}\rangle }_{v}\int e^{iQ\cdot r}\gamma \left(r\right)d^{3}r. \end{array}$$ (96)

The first term is a *Q*-independent background, and the second is the forward coherent scattering, which usually is masked by a beam stop. The Fourier transform of *γ*(***r***) in the last term describes the scattering caused by the spatial variation of the microstructure. Equation (96) is the basic law of small angle neutron scattering.

A typical class of materials in SANS studies consists of two or more homogeneous phases separated by sharp interfaces and partitioned into multiply-connected volume compartments. For such systems the scattering-length density can be represented as

$$\eta \left(r\right)=\sum_{n}{\bar{b}}_{n}\rho_{n}\left(r\right),$$ (97)

where

$$\rho_{n}\left(r\right)=\sum_{icn}\delta \left(r-R_{i}\right),$$ (98)

and ${\bar{b}}_{n}$ is its coherent scattering-length, which can be computed as a cellular average for polyatomic phases. The component densities satisfy

$${\langle \rho_{n}\rangle }_{v}=\frac{N}{V}\phi_{n},$$ (99)

where *ϕ*_n_ is the volume fraction of the *n*-th phase, and also have the properties,

$$\rho_{n}\left(r\right)\rho_{m}\left(r\right)\{\begin{array}{ll} 0 & \mathrm{for}\;m\neq n,\\ \frac{N}{V}\rho_{n}\left(r\right) & \mathrm{for}\;m=n. \end{array}$$ (100)

The first expresses the spatial partitioning of the phases, while the second is the normalized idempotency condition. It then follows that

$${\langle {\left(\Delta \eta \right)}^{2}\rangle }_{v}={\left(\frac{N}{V}\right)}^{2}\left[\langle b_{\mathrm{coh}}^{2}\rangle -{\langle b_{\mathrm{coh}}\rangle }^{2}\right],$$ (101)

where

$$b_{\mathrm{coh}}=\sum_{n}\phi_{n}{\bar{b}}_{n}.$$ (102)

For the special but important case of two phases, this reduces to the symmetrical formula

$${\langle {\left(\Delta \eta \right)}^{2}\rangle }_{v}=\phi \left(1-\phi \right){\left(\eta_{1}-\eta_{2}\right)}^{2},$$ (103)

where *ϕ* is the volume fraction of either one of the phases, and *η*_1_ and *η*_2_ are the scattering-length densities.

### 3.5 Inelastic Scattering

In Eq. (53) the non-elastic contribution, $S_{\alpha }^{\mathrm{ne}}\left(Q,\omega \right),$ may describe *in*elastic scattering, *quasi*elastic scattering, Sec. 3.6, or both, depending on the system being studied. Inelastic scattering means energy-resolved scattering *not* centered on *ω* = 0. Coherent inelastic scattering typically consists of well-defined lines riding on a diffuse, or structureless, *ω*-dependent background. The lines result from resonant scattering of the neutron by long-lived elementary excitations of the system, such as phonons and magnons (in magnetic materials) and impurity atom vibrations in host-guest systems. Diffuse coherent inelastic scattering is produced by scattering from multiple, or non-elementary, excitations. Energy-integrated inelastic scattering is usually the main source of the thermal diffuse background in structure determinations using energy-insensitive detection methods. Incoherent inelastic scattering measures excitation densities of states, to a first approximation.

#### 3.5.1 Scattering by Phonons

Scattering by phonons provides the prototype for neutron inelastic scattering. Squires [18] gives a lucid treatment. To calculate the intermediate scattering function, Eq. (27), resolve the nuclear position operator into the equilibrium coordinate and a time-dependent small displacement,

$$R_{j}\left(t\right)=R_{j}^{\mathrm{eq}}+u_{j}\left(t\right).$$ (104)

Then, using the phonon representation of the displacement operator, one can derive (with some tedium) the basic identity of the harmonic approximation,

$$\begin{array}{l} \langle e^{-iQ\cdot R_{i}\left(0\right)}e^{iQ\cdot R_{j}\left(t\right)}\rangle =\\ \;e^{-iQ\cdot \left(R_{i}^{\mathrm{eq}}-R_{j}^{\mathrm{eq}}\right)}e^{-2W}e^{2W_{ij}}\left(Q,t\right), \end{array}$$ (105)

where

$$W_{ij}\left(Q,t\right)=\frac{1}{2}\langle Q\cdot u_{i}\left(0\right)Q\cdot u_{j}\left(t\right)\rangle ,$$ (106)

and

$$W=W_{ii}\left(Q,0\right).$$ (107)

The ubiquitous exponential, exp(−2*W*), is the *Debye-Waller factor*. It is a long-standing convention to leave the ***Q***-dependence of the *W* function implicit. A concrete formula for *W* follows from Eq. (113) in the next section. Although correct quantum mechanically, the result in Eq. (105) actually can be obtained more directly by treating ***Q*** · ***u****_j_* (*t*) as a classical Gaussian random variable, *ζ*, with zero mean, and by using the concommitant rule:

$$\langle e^{\zeta }\rangle =e^{1/2\langle \zeta^{2}\rangle }.$$

The Taylor expansion of the final exponential in Eq. (105) leads to a systematic development of the inelastic scattering function, $S_{\alpha }^{\mathrm{inel}}\left(Q,\omega \right).$ The first term (unity) gives the elastic scattering from the equilibrium structure, as in Eq. (56). The next term—the first *t*-dependent one—describes *one-phonon* scattering, while the remainder produces *multi-phonon* scattering. For a Bravais lattice the result for coherent one-phonon scattering turns out to be:

$$\begin{array}{c} S_{\mathrm{coh}}^{1-\mathrm{ph}}\left(Q,\omega \right)=\frac{{\left(2\pi \right)}^{3}}{\Omega_{0}}\frac{\hbar e^{-2W}}{2M}\sum_{q,s,k}\frac{{\left[Q\cdot \epsilon_{s}\left(q\right)\right]}^{2}}{\omega_{s}\left(q\right)}\\ \{\left[n_{s}\left(q\right)+1\right]\delta \left(\omega -\omega_{s}\left(q\right)\right)\delta \left(Q-q-K\right)\\ +\left[n_{s}\left(q\right)\right]\delta \left(\omega +\omega_{s}\left(q\right)\right)\delta \left(Q+q+K\right)\}. \end{array}$$ (108)

In this formula ***M*** is the nuclear mass, *Ω*_0_ is the volume of the lattice unit cell, and ***K*** is a reciprocal lattice wavevector (often denoted as 2*πτ*, where ***τ*** is a reciprocal lattice vector). The phonon modes of wavevector ***q*** and polarization *s* have frequencies *ω_s_*(***q***), polarization vectors ***∊****_s_*(***q***) and thermal occupations *n_s_*(***q***). The two terms in Eq. (108) describe, respectively, scattering with the emission of a phonon—the neutron is *down*-scattered by energy *ℏω_s_*(***q***)—and scattering with the absorption of a phonon—the neutron is *up*-scattered by energy *ℏω_s_*(***q***). In each case the scattering satisfies the kinematical constraints

$$\frac{\hbar^{2}}{2m}\left(k_{0}^{2}-k^{2}\right)=\pm \hbar \omega_{s}\left(q\right)$$ (109)

and

$$k_{0}-k=K\pm Q.$$ (110)

For *incoherent* one-phonon scattering, the scattering function can be obtained from Eq. (108) by making the formal replacement,

$$\frac{{\left(2\pi \right)}^{3}}{\Omega_{0}}\delta \left(Q\mp q-K\right)\underset{\to }{\mathrm{inc}}1.$$

This leads to

$$\begin{array}{l} S_{\mathrm{inc}}^{1-\mathrm{ph}}\left(Q,\omega \right)=\\ \;\frac{3N\hbar e^{-2W}}{2M}\frac{{\langle {\left[Q\cdot \epsilon_{s}(q)\right]}^{2}n_{s}\left(q\right)\rangle }_{\omega }}{\omega }N\left(\omega \right), \end{array}$$ (111)

where *N*(*ω*) is the one-phonon density of states and 〈…〉*_ω_* denotes the average over the surface in reciprocal space on which *ω_s_*(***q***) = *ω*.

For multi-phonon processes the corresonding kinematical relations are not mode-specific, and diffuse *ω*-dependent scattering generally is the result. Often the multi-phonon background must be estimated in order to achieve a reliable analysis of one-phonon scattering. Occasionally multi-phonon scattering may even appear in the guise of “broadened” one-phonon scattering in complicated spectra.

Inelastic scattering is also used to measure spin-wave spectra in magnetic materials. The kinematical constraints for 1-*magnon* scattering are the same as in Eq. (110). Determination of local vibrations in host-guest systems is another important application of inelastic scattering. For example, in hydrogen in metals, the point symmetry of occupied interstitial sites usually is revealed simply by assignment of the intensities produced by vibrational modes having signature degeneracies.

#### 3.5.2 Thermal Diffuse Scattering

With the material now at hand, we continue the description of thermal diffuse scattering begun in Sec. 3.2. In general the energy-integrated non-elastic scattering that constitutes thermal diffuse scattering incorporates both quasielastic and inelastic scattering. Here, however, we only consider the inelastic contribution from phonon scattering. Thus using Eq. (105) in Eqs. (28) and (62), and recalling Eq. (71), one gets

$$\begin{array}{l} S_{\mathrm{coh}}^{\mathrm{ne}}\left(Q\right)=1-e^{-2W}+\frac{e^{-2W}}{N}\\ \;\sum_{i\neq j}e^{-iQ\cdot \left(R_{i}^{\mathrm{eq}}-R_{j}^{\mathrm{eq}}\right)}\left(e^{2W_{ij}\left(Q,0\right)}-1\right), \end{array}$$ (112)

where *W_ij_*(***Q***,*t*) was defined in Eq. (106). Explicitly, for *t* = 0,

$$\begin{array}{l} W_{ij}\left(Q,0\right)=\frac{\hbar }{4MN}\sum_{q,s}\frac{{\left[Q\cdot \epsilon_{s}\left(q\right)\right]}^{2}}{\omega_{s}\left(q\right)}\\ \;\left[2n_{s}\left(q\right)+1\right]\cos\left[q\cdot \left(R_{i}^{\mathrm{eq}}-R_{j}^{\mathrm{eq}}\right)\right]. \end{array}$$ (113)

The formula for *W*, Eq. (107), is obtained by setting *i* = *j* here. For an Einstein model the thermal displacements on different sites are uncorrelated, so that *W_ij_*(***Q***,0) = 0 for *i* ≠ *j*. In this case, therefore, the term in Eq. (112) with the sum over sites vanishes identically, and the thermal diffuse background is 1-exp(−2*W*). Notice that in the opposite extreme, if *W_ij_*(***Q***,0) were independent of the distance between sites, the summation in Eq. (112) would produce Bragg peaks, as in $S_{\mathrm{coh}}^{\mathrm{el}}\left(Q\right)$. Instead, $W_{ij}\left(Q,0\right)\to 0\;\mathrm{as}\;\left|R_{i}^{\mathrm{eq}}-R_{j}^{\mathrm{eq}}\right|\to \infty$ and this fall-off moderates the site summation. However, *W_ij_*(***Q***,0) decays slowly with distance, which leads to a weakened expression of the reciprocal lattice structure in the thermal diffuse scattering. This is seen by expanding exp[2*W_ij_*(***Q***,0)], in analogy to the phonon expansion of $S_{\mathrm{coh}}^{\mathrm{inel}}\left(Q,\omega \right)$. Indeed, one can obtain the 1-phonon thermal diffuse scattering directly by integrating Eq. (108) with respect to *ω*.This gives

$$\begin{array}{l} S_{coh}^{1-\mathrm{ph}}\left(Q\right)=\frac{{\left(2\pi \right)}^{3}}{\Omega_{0}}\frac{\hbar^{e-2W}}{2M}\\ \;\sum_{s,K}{\left[Q\cdot \epsilon_{s}\left(\tilde{q}\right)\right]}^{2}\frac{\left[2n_{s}\left(\tilde{q}\right)+1\right]}{\omega_{s}\left(\tilde{q}\right)}, \end{array}$$ (114)

where

$$\tilde{q}=Q-K$$

for each ***K*** in the sum. Thus the acoustic phonon branches give rise to algebraic singularities coincident with the Bragg peaks, since $\omega_{s}\left(\tilde{q}\right)=c_{s}\bar{q}$ for small $\left|\tilde{q}\right|.$ Specifically, for $\tilde{q}\to 0$ near a particular ***K*** and at temperatures such that $\hbar \omega \left(\tilde{q}\right)\ll k_{B}T,$ then

$$S_{\mathrm{coh}}^{1-\mathrm{ph}}\left(Q\right)\propto {\left|Q-K\right|}^{2},$$ (115)

since $n_{s}\left(\tilde{q}\right)\sim k_{B}T/\hbar \omega_{s}\left(\tilde{q}\right)$. On the other hand, if $\hbar \omega_{s}\left(\tilde{q}\right)\gg k_{B}T,$ then

$$S_{\mathrm{coh}}^{1-\mathrm{ph}}\left(Q\right)\propto {\left|Q-K\right|}^{-1},$$ (116)

Although these results are derived in the harmonic approximation, they correctly imply that soft modes in plastic crystals also produce significant thermal diffuse scattering near ***Q*** = ***K*** *+* ***q***_c_, where *ω_s_*(***q***_c_) = 0 at the softening temperature.

Historically, before the ascendancy of inelastic neutron scattering, x-ray thermal diffuse scattering was the chief means of measuring phonon dispersion in solids by fitting lineshapes with expressions like Eq. (114).

### 3.6 Quasielastic Scattering

Quasielastic scattering is energy-resolved scattering centered on *ω* = 0 and is the result of neutrons interacting with purely dissipative excitations, which can be viewed as motions at imaginary frequencies. Usually this means scattering by diffusing nuclei. Typically, quasielastic and inelastic scattering are well-separated from each other, since vibrational angular frequencies tend to be much larger than diffusive jump rates. Sometimes, however, analysis of the scattering near *ω* = 0 into its elastic, quasielastic and inelastic components is not easy.

A prototype for quasielastic neutron scattering is incoherent quasielastic scattering from hydrogen in metals. At high temperatures the correlation function, *G*_inc_(***r***,*t*) in Eq. (30), is well-approximated by the classical limit,

$$\begin{array}{l} G_{\mathrm{inc}}^{\mathrm{class}}\left(r,t\right)=P_{\mathrm{self}}\left(r,t\right)\\ \;=\langle \delta \left(r+R_{0}\left(0\right)-R_{0}\left(t\right)\right)\rangle , \end{array}$$ (117)

which is the finite-*t* generalization of Eq. (58). Application of jump diffusion theory to the calculation of *P*_self_(*r*,*t*) then leads to the Lorentzian quasielastic lineshape,

$$S_{\mathrm{inc}}\left(Q,\omega \right)=\frac{\Gamma \left(Q\right)/\pi }{\omega^{2}+\Gamma {\left(Q\right)}^{2}},$$ (118)

where *Γ*(***Q***) depends on the diffusion constant and on the structure visited by the diffusing particle. Incoherent quasielastic scattering coexists with incoherent elastic scattering given by Eq. (57), which is called the *elastic incoherent structure factor* in the concerned literature. This “EISF” is proportional to the reciprocal of *Ω*_self_, the volume accessible to the diffusing particle. Thus, the less localized the self-diffusion, the smaller the weight of incoherent elastic scattering.

Coherent quasielastic scattering, which was alluded to in Sec. 3.3, is important in fluid systems, indeed wherever diffusive transport of coherently scattering nuclei occurs. Quasielastic scattering also is produced by diffusive rotational modes in plastic crystals and caged systems. See [4] for an extensive treatment of quasielastic scattering.

## 4. Neutron Refraction and Reflection

The Born approximation neglects interference between the incident and scattered waves. This usually is justified in standard beam-target geometries for scattering at wide angles from small samples. These interactions become important, however, as scattering becomes concentrated into the forward direction. Then the sharp distinction between incident and scattered beams within the scattering medium is lost, and it is necessary to solve the Schrödinger equation (“dynamical” scattering) without resort to the Born approximation (“kine-matical” scattering).

An important problem where dynamical scattering theory is required is a neutron beam incident on a semi-infinite scattering medium having a smooth, planar boundary, say at *z* = 0. The Schrödinger equation for this case is

$$\frac{-\hbar^{2}}{2m}\nabla^{2}\psi \left(r\right)+V\left(r\right)\psi \left(r\right)=E_{0}\psi \left(r\right),$$ (119)

where $E_{0}=\hbar^{2}k_{0}^{2}/2m$, and the potential is the step function,

$$V\left(r\right)=\{\begin{array}{cc} 0, & \;z>0\\ {\langle V\rangle }_{\mathrm{cell}} & \;z\leq 0 \end{array}.$$ (120)

The effective scattering potential in the medium is the average of *V*(***r***) over the crystalline unit cell. This is a good approximation for neutrons beyond the Bragg cutoff. In detail,

$${\langle V\rangle }_{\mathrm{cell}}=\frac{2\pi \hbar^{2}}{m}\rho {\langle b_{\mathrm{coh}}\rangle }_{\mathrm{cell}},$$ (121)

where *ρ* = *N/V*, and *b*_coh_ is arithmetically averaged over the cell. The trial solution is

$$\psi \left(r\right)=\{\begin{array}{cc} e^{ik_{0}\cdot r}+u_{r}e^{ik_{r}\cdot r} & \;z>0\\ u_{m}e^{ik_{m}\cdot r} & \;z\leq 0 \end{array}.$$ (122)

where *k*_r_ = *k*_0_. The subscript “r” denotes the reflected beam, while “m” refers to the wave within the scattering medium. The angle of incidence *ϕ* and the angles of the reflected and refracted waves *ϕ*_r_ and *ϕ*_m_, respectively, are conventionally measured from the flat in neutron optics. Since the model potential is everywhere independent of *x* and *y*, the wavevectors *k*_0_, ***k***_r_ and *k*_m_ have equal components parallel to the surface. Thus the reflection is specular, *ϕ*_r_ = *ϕ*. The boundary conditions at *z* = 0 are

$$\begin{array}{c} 1+u_{r}=u_{m},\;\mathrm{and}\\ \left(1-u_{r}\right)k_{0}\;\sin\;\phi =u_{m}k_{m}\sin\;\phi_{m}. \end{array}$$ (123)

It follows easily from Eq. (122) in Eq. (119) that

$$\frac{\hbar^{2}k_{0}^{2}}{2m}=\frac{\hbar^{2}k_{m}^{2}}{2m}+{\langle V\rangle }_{\mathrm{cell}}.$$ (124)

This gives the index of refraction of the scattering medium,

$$n=\frac{k_{m}}{k_{0}}=\sqrt{1-\frac{{\langle V\rangle }_{\mathrm{cell}}}{E_{0}}}.$$ (125)

The amplitude of the reflected wave works out to

$$u_{r}=\frac{\sin\phi -\sqrt{\sin^{2}\phi -\sin^{2}\phi_{c}}}{\sin\phi +\sqrt{\sin^{2}\phi -\sin^{2}\phi_{c}}},$$ (126)

where *ϕ*_c_ is the critical angle,

$$\phi_{c}=\arcsin\sqrt{1-n^{2}}.$$ (127)

For *ϕ* ≤ *ϕ*_c_, Eq. (126) shows that |*u*_r_| = 1, which indicates that the incident wave is totally reflected. In this case the normal component of ***k***_m_ is imaginary, and the wave in the medium is evanescent. When *ϕ* > *ϕ*_c_, *k*_m_ is real, and its direction is given by Snell’s law. The index of refraction in Eq. (125) can be written as

$$n\approx 1-\frac{\lambda_{0}^{2}}{2\pi }\eta ,$$ (128)

where *η* = *ρ*〈*b*_coh_〉_cell_ is the average scattering length density of the medium and generally is positive. Typically *n* = 1 − *O*(10^−5^) for thermal neutrons, which gives critical angles

$$\phi_{c}\approx \lambda_{0}\sqrt{\frac{\eta }{\pi }}$$ (129)

of only fractions of a degree. The longer wavelengths of cold neutrons give substantially larger critical angles, which are required for practical applications of neutron guides. For media in which *η* < 0, the index of refraction is greater than unity, *ϕ*_c_ is imaginary, and the incident wave is never totally reflected (except at *ϕ* = 0).

The scattering wavevector for neutron reflection is defined as

$$Q=k_{0}-k_{r},$$ (130)

so that *Q* = 2*k*_0_ sin *ϕ*. The neutron reflectivity, *R* = |*u*_r_|^2^, then is obtained from Eq. (126) as

$$R\left(Q\right)={\left[\frac{Q-\sqrt{Q^{2}-Q_{c}^{2}}}{Q+\sqrt{Q^{2}-Q_{c}^{2}}}\right]}^{2},$$ (131)

where *Q*_c_ = 2*k*_0_ sin *ϕ*_c_. This is equivalent to the Fresnel formula for reflectivity in optics. Thus, *R*(*Q*) = 1 for *Q* ≤ *Q*_c_, while for *Q* ≥ *Q*_c_, the reflectivity falls as

$$R\left(Q\right)∼\frac{16\pi^{2}\eta^{2}}{Q^{4}}.$$ (132)

The asymptotic behavior in Eq. (132) is identical to that derived from the Born approximation for the potential in Eq. (120) when d*σ*/d*Ω*, Eq. (60), is converted to the reflectivity, an observation made only recently by Sinha, et al. [16]. The reflection laws from heterogeneous and nonuniform surfaces and films depart significantly from Eq. (131) and are sensitive to model details. For expositions of neutron reflectometry and optics, see [9], [12], and [15].
